# Tregs levels and phenotype modifications during Amyotrophic Lateral Sclerosis course

**DOI:** 10.3389/fimmu.2024.1508974

**Published:** 2025-01-08

**Authors:** Elisabetta Zucchi, Federico Banchelli, Cecilia Simonini, Sara De Biasi, Ilaria Martinelli, Giulia Gianferrari, Domenico Lo Tartaro, Andrea Cossarizza, Roberto D’Amico, Jessica Mandrioli

**Affiliations:** ^1^ Neuroscience PhD Program, University of Modena and Reggio Emilia, Modena, Italy; ^2^ Department of Neurosciences, Ospedale Civile di Baggiovara, Azienda Ospedaliero-Universitaria di Modena, Modena, Italy; ^3^ Department of Medical and Surgical Sciences for Children and Adults, University of Modena and Reggio Emilia, Modena, Italy; ^4^ National Institute for Cardiovascular Research, Bologna, Italy; ^5^ Unit of Statistical and Methodological Support to Clinical Research, Azienda Ospedaliero-Universitaria, Modena, Italy; ^6^ Department of Biomedical, Metabolic and Neural Sciences, University of Modena and Reggio Emilia, Modena, Italy

**Keywords:** ALS, T regulatory cell, ALSFRS-r, FVC (forced vital capacity), cholesterol, monocyte

## Abstract

**Introduction:**

T regulatory cells (Tregs) inversely correlate with disease progression in Amyotrophic Lateral Sclerosis (ALS) and fast-progressing ALS patients have been reported to exhibit dysfunctional, as well as reduced, levels of Tregs. This study aimed to evaluate the longitudinal changes in Tregs among ALS patients, considering potential clinical and biological modifiers of their percentages and concentrations. Additionally, we explored whether measures of ALS progression, such as the decline over time in the revised ALS Functional Rating Scale (ALSFRS-r) or forced vital capacity (FVC) correlated Treg levels and whether Treg phenotype varied during the course of ALS.

**Methods:**

Total Tregs (detected by CD3, CD4, FoxP3, CD25, and CD127) were quantified at five time points over 54 weeks in 21 patients in the placebo arm of the RAP-ALS trial; next they were characterized for the expression of surface markers including CD38, CD39, CXCR3, and PD1. Repeated measures mixed models were used to analyze the longitudinal course of Tregs, considering potential associations with other clinical and laboratory characteristics. Correlations between ALSFRS-r or FVC and Tregs over time were similarly investigated.

**Results:**

Our study showed that Treg levels did not change significantly on average during the observation period in our ALS cohort. However, PD1+Tregs decreased and CD39+Tregs increased over time. Male sex and cholesterol levels were associated with increasing Tregs (%) over time, while monocytes positively affected Treg concentrations. Treg concentrations showed a modesty association with FVC decline but were not associated with ALSFRS-r decline.

**Discussion:**

Treg levels remained stable during the ALS observation period and were not significantly associated with ALSFRS-r variations, suggesting that Treg numbers alone may have limited utility as a pharmaco-dynamic biomarker for ALS trials. However the observed changes in Treg phenotypes, such as the decrease in PD1+Tregs, indicate that phenotypic variations may warrant further investigation for their potential role in ALS progression and therapeutic targeting.

## 1 Introduction

Amyotrophic Lateral Sclerosis (ALS) is a rare neurogenerative disorder affecting the upper and lower motor neurons, with a global lifetime risk of approximately one in 350 people (1, 2). Notwithstanding death or permanent ventilation results in three to five years since the onset of disease, there is wide heterogeneity in clinical presentation, and different phenotypes, with prognostic repercussions, are recognized in ALS (2). While the mechanisms beneath the motor neuronal degeneration have not been fully elucidated, multiple lines of evidence point to non-cell autonomous processes contributing to the damage to the motor neurons (3).

Crucial to the neuroinflammatory modulation, T regulatory cells (Tregs), a subset of T cells characterized by expression of CD4+, FoxP3+, CD25+, are known as potential modifiers of disease progression in Amyotrophic Lateral Sclerosis (ALS) (4). Tregs are central to immune homeostasis, by regulating the delicate equilibrium between immune activation and tolerance (5), with specific roles in preventing autoimmune conditions (6), controlling systemic inflammation (7) and contrasting immunosurveillance for tumor expansion (8). In SOD1-mutated mice with ALS, increased concentrations of Tregs were observed during the early phases of the disease and later decreased, consistent with an M1-like toxic microglia environment switch into the spinal cord; in this model passive transfer of functional Tregs prolonged survival (9). Moreover, in fast-progressing patients with ALS, reduced (9, 10) and dysfunctional Tregs (11) were found compared to slowly progressing patients, and FoxP3 expression, their master regulator, inversely correlated with disease progression and survival.

Based on this evidence, several trials have attempted to expand Tregs and restore their function as a targetable mechanism for slowing the course of ALS (12–14), by tuning down the pro-inflammatory systemic immune background detected in most ALS patients (4). While some randomized controlled trials (RCTs) are currently ongoing, setting as a primary objective to increase Tregs or FoxP3 expression compared to baseline values (14), many of the completed trials could not reach this biological outcome.

However, while some preliminary data from SOD1-mutated mice highlighted a disease progression-dependent decrease in peripheral Tregs during active phases of ALS (9), longitudinal studies assessing Tregs number in ALS patients are lacking, and interpretation of Treg variations compared to placebo in clinical trials might be challenging. In line with the continuously growing knowledge about more complex Treg phenotypes, tissues of origin, or capabilities to adapt to different inflammatory challenges, other demographic or biological factors may modify Treg number or functions and should be considered for the sake of efficacy analysis in the design of clinical trials for ALS therapy.

The primary objective of this study was to evaluate the longitudinal trajectories of Tregs in patients with ALS, including possible modifiers of their percentages or concentrations. Secondly, the relationship between the decline in ALSFRS-r or forced vital capacity (FVC) with Tregs and the longitudinal trajectories of Treg subpopulations, as defined by expression of specific surface markers, was explored in ALS patients.

## 2 Methods

### 2.1 Study participants

For this study, we considered patients who were enrolled in the RAP-ALS clinical trial and allocated to placebo arm. These patients (21 subjects) were treated with Riluzole but were not on other experimental drugs and underwent the study procedures which included serial serum sampling from baseline to week 54 as previously reported (15). The study design, subject selection, and participants in RAP-ALS clinical trial have been reported elsewhere (15, 16). Briefly, 63 patients with definite, clinically probable or probable with laboratory support ALS according to revised El Escorial Criteria, within 18 months since onset of motor symptoms were recruited from 2017 through 2020. Regularly scheduled visits at week 4, 8, 12, 18, 30, 42 and 54 were performed with blood samples collection at week 8, 18, 30, 42 and 54.

The revised form of the ALS Functional Rating Scale (ALSFRS-r), weight, body mass index (BMI), vital parameters, neurological examination and forced vital capacity (FVC) were assessed at baseline (week 0), and at weeks 4, 8, 12, 18, 24, 30, 42, 54. Weight change was calculated as the actual weight recorded at each visit minus the weight before the onset of disease.

### 2.2 Standard protocol approvals, registrations, and patient consents

Ethical approval was obtained by Ethical Committee of the coordinating center (Comitato Etico Provinciale di Modena) in May 2017 (file number 95/17) and written informed consent was obtained from all the participants.

### 2.3 Tregs and other laboratory assessments

**Figure 1:**
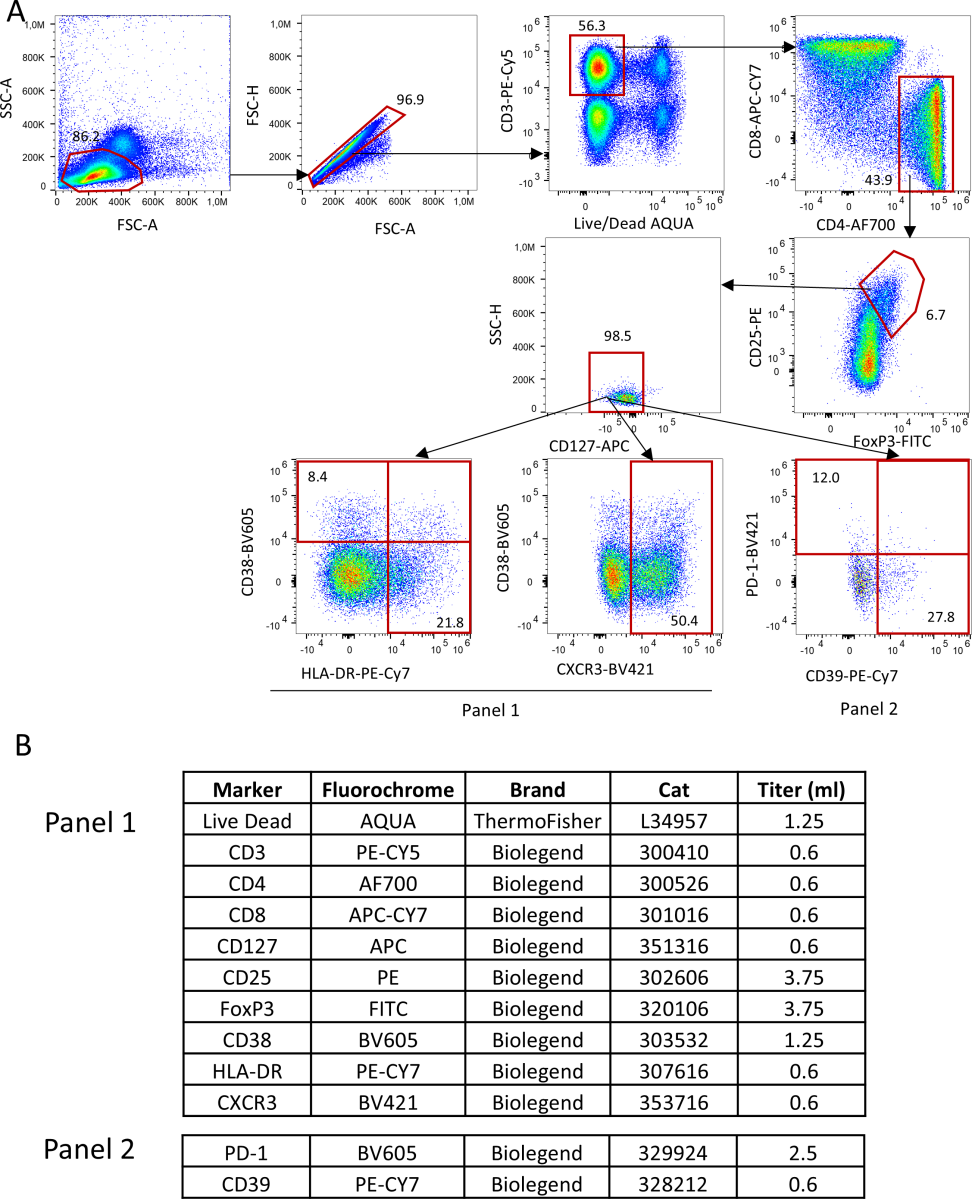
Gating strategy used to identify Tregs and their subpopulations. **(A)** Lymphocytes were identified by physical parameters, doublets were removed and alive CD3+ T cells were selected accordingly. In this population, CD4+ T were selected and within this, those who were CD25++, FoxP3+ and CD127- were defined as Tregs. In this population, by using two different panels (which shared the same backbone, such as Live Dead, CD3, CD4, CD8, CD127, CD25,FoxP3; all reported in **(B)**, Tregs expressing HLA-DR, CD38, CXCR3, PD-1 and CD39 were identified.

All the biological procedures related to RAPALS study, including Tregs number and function assessment, have been previously reported (15, 16). In brief, immunophenotype of Treg cell subpopulations were performed by polychromatic flow cytometry at baseline and week 8, 18, 30, 54 as shown in (**Figure 1**). Freshly isolated PBMC were washed and stained with commercially available monoclonal antibodies (mAb) directly conjugated with different fluorochromes. For T regulatory cells phenotype, the following markers, besides viability marker (AQUA Live Dead) were used: anti-CD3-Pe-Cy5, -CD4-AF700, CD8-APC-Cy7, CD25-PE, CD127-APC-Cy7, HLA-DR-PE-Cy7, CXCR3-BV421, CD38-BV605, PD-1-BV605, CD39-BV421. Treg cells were identified as FoxP3+, CD25++, CD127- cells within CD3+CD4+ T cells. A first activation/homing panel was set with the following markers for CD4+ and CD8+ T cells, Tregs included: CD38 and CXCR3. CD38 was included for its role in modulating immune suppression and its association with NAD+ metabolism (17), which could influence Treg function in the inflammatory environment. CXCR3 is noted for its involvement in Treg migration to inflamed tissues (18).

A second metabolic/exhaustion/activation panel was similarly set for Tregs and CD4+, CD8+ T cells according to the expression of PD1 and CD39 (**Figure 1**). CD39 is a key ectoenzyme contributing to the generation of adenosine, an anti-inflammatory molecule, potentially reflecting a compensatory mechanism in response to neuroinflammation (19). PD-1 is mentioned as a marker of Treg exhaustion, whose decrease over time may indicate a loss of immunosuppressive capacity (20).

Tregs and their subpopulations were expressed as percentages over total Tregs in the case of Tregs subpopulations, and as percentages over total CD4 T cells, as assessed by flow cytometric analysis at the central University lab, or as concentrations (number of cells per microliter), in the case of total Tregs. These concentrations were obtained by calculating the proportion of Treg/total CD4 T cells from the percentage obtained at the University lab by the gating strategy and the total number of lymphocytes as assessed by standard labs of each MND center.

We were unable to include five measurements for all patients due to dropouts during the RAP-ALS trial (15). Additionally, logistical challenges in collecting and processing fresh samples, such as the limited availability of biological material, further affected our ability to obtain five measurements for every patient.

Neurofilament heavy chain (NfH) and light chain (NfL) were analyzed in plasma by Ella Automated Immunoassay System (Simple Plex Human NF Cartridge, R&D System, Minneapolis, MN), according to the manufacturer’s instructions and as already reported (21, 22).

Standard blood exams including hematology, biochemistry (creatinine, urea, uric acid, total cholesterol, LDL cholesterol, triglycerides, ALT, AST, GGT) and urinalysis were performed by certified local laboratories at week 4, 8, 12, 18, 24, 30, 42 and 54.

### 2.4 Model design for Tregs variations

To explain the variations of Tregs during time, the effect of several demographic, clinical and laboratory variables was assessed by considering all these explanatory variables: time since onset of disease, ALSFRS-r, FVC, age at onset of disease, site of onset (bulbar vs spinal), weight loss since onset of disease, sex, BMI, albumin, creatinine, C-reactive protein (CRP), uric acid, total cholesterol, triglycerides, monocytes, neutrophil-to-lymphocytes ratio (NLR), and serum neurofilament light (NfL). ALSFRS-r and FVC were chosen to investigate if their decline could explain Tregs variations over time. Since Tregs may be affected by sexual hormones, adiposity and aging, we included sex, BMI, weight change, which is also a prognostic factor for ALS (23), and age at onset, as other explanatory variables. The site of onset, besides being a crucial independent prognostic factor for ALS, was added to correct for different trajectories of ALSFRS-r or FVC decline, as bulbar onset or bulbar phenotype patients might show steeper declines during the early phases of ALS or have more difficulties in performing functional respiratory tests (24). Regarding lab values, we chose CRP as a general marker of systemic inflammation, monocytes and NLR to investigate other aspects of systemic inflammation with novel roles recently linked to ALS progression (25–28), albumin to account for nutritional status, creatinine as a marker of renal function and a known biomarker of progression in ALS (29), total cholesterol and triglycerides as markers of lipid metabolism possibly related to Treg cells (30), uric acid for protein metabolism, serum NfL to investigate possible relations between the pace of neurodegeneration with Tregs variations.

### 2.5 Statistical methods

Characteristics of patients were described using mean, standard deviation and range for numerical variables and using absolute and percentage frequencies for categorical variables.

The association between the candidate explanatory variables (demographics, anthropological measures, clinical characteristics, neurofilaments, and laboratory values over time) and the outcomes of interest (Tregs percentages and concentrations, ALSFRS-r and FVC) measured at each time point was assessed using generalized linear mixed models. Since Tregs percentages and concentrations may explain different immunological effects, they were considered as separate outcome variables during the analyses.

Firstly, unadjusted models including time from onset as the only independent variable were estimated, to describe the observed linear trend over time of Tregs, ALSFRS-r and FVC. Secondly, multivariable models including all candidate independent variables were estimated, to describe which demographic, clinical and laboratory characteristics were associated with the outcomes.

All independent variables were time-varying except for age, sex, and site of onset. When analyzing ALSFRS-r and FVC as the dependent variables, albumin, CRP, uric acid, total cholesterol, monocytes, creatinine, triglycerides and NLR were not considered as independent variables.

An individual random intercept term was included in all models to allow the average value of the outcome to vary between patients. To allow the outcome slope to vary over time between patients, a random slope term was included in all unadjusted models and, if it improved the Bayesian information criterion (BIC) value, also in multivariable models.

Thirdly, we investigated if other specific Treg subpopulations could be influenced by ALSFRS-r, FVC and the other individual characteristics measured at each time point. The following cell markers were used to classify Treg subpopulations: CD38, CXCR3, CD39, PD1.These markers of Treg activity were considered as additional dependent variables, all measured as percentage over total Tregs, percentage over CD4 T cells or concentration.

Tregs percentages and concentration (including Tregs subpopulation) were analyzed using a multiplicative effect generalized linear mixed model with gamma distribution and log link function, whereas ALSFRS-r and FVC were analyzed using an additive effect linear mixed model with gaussian distribution and identity link function. The choice of the models was based on the BIC comparing several alternative models (including gaussian, log-gaussian, inverse gaussian, gamma and beta models with additive or multiplicative effects), as well as on goodness-of-fit measures and on visual inspection of the shape and skewness of the outcomes distributions.

Results for multiplicative effect models were reported as the mean ratio (MR), whereas results for additive effect models were expressed as the mean difference (MD). Uncertainty in estimated was reported as the 95% confidence interval (CI).

Missing values in the laboratory explanatory variables were imputed for each patient by using the linear trend criterion, bounded within the minimum and maximum observed values for that individual. For all models, listwise deletion was applied for records with missing values in the other independent variables.

Analyses were performed with R 4.3.2 (The R Foundation for Statistical Computing, Wien). Statistical significance was set at p < 0.05, and results with p < 0.10 were considered of potential interest.

## 3 Results

### 3.1 Tregs natural history in ALS patients

All 21 patients who were allocated to the placebo arm in the RAP-ALS trial were included in this analysis. Their clinical features are summarized in **Table 1** and the baseline values of total Treg counts, together with Tregs identified by phenotypic markers, are described in **Supplementary Table 1**. Female patients experienced more severe weight loss at baseline, compared to the weight before the onset of the disease, and were more likely to present with bulbar onset.

**Table 1:** Clinical features of ALS patients included in the analysis of Tregs longitudinal variations.

Clinical features	Females (n=8)	Males (n=13)	Total (n=21)
Age at baseline (years)	59.30 (10.05)	53.25 (14.23)	55.55 (12.88)
Age at onset (years)	58.39 (10.09)	52.18 (14.03)	54.54 (12.78)
Spinal site of onset	4 (50.0%)	11 (84.6%)	15 (71.4%)
Weight at baseline (kg)	59.88 (9.30)	78.24 (9.56)	71.24 (12.98)
BMI at baseline (kg/m^2^)	24.16 (3.16)	24.85 (2.21)	24.59 (2.56)
Weight loss at baseline (kg)	2.00 (2.73)	0.53 (3.80)	1.09 (3.44)
Time since first ALS symptom (months)	10.5 (4.41)	12.38 (4.15)	11.67 (4.25)
ALSFRS-r score at baseline	38.63 (5.40)	38.62 (5.56)	38.62 (5.36)
Disease progression rate at baseline	1.28 (1.28)	0.84 (0.66)	1.00 (0.94)
FVC at baseline (%)	91.05 (19.49)	97.5 (13.16)	95.04 (15.72)
Kings’ staging at baseline	1.75 (0.46)	1.77 (0.44)	1.76 (0.44)
Albumin at baseline (g/dL)	4.69 (0.70)	4.52 (0.34)	4.58 (0.50)
CRP at baseline (mg/dL)	0.79 (1.34)	0.66 (0.77)	0.71 (1.00)
Uric acid at baseline (mg/dL)	3.84 (0.82)	5.47 (1.12)	4.85 (1.28)
Total cholesterol at baseline (mg/dL)	251.00 (39.79)	196.46 (20.86)	217.24 (39.39)
Monocytes at baseline (counts/mm^3^)	0.49 (0.13)	0.52 (0.14)	0.51 (0.13)
Creatinine at baseline (mg/dL)	0.70 (0.10)	0.79 (0.16)	0.75 (0.15)
Triglycerides at baseline (mg/dL)	95.38 (28.69)	110.46 (43.72)	104.71 (38.62)
NLR at baseline	2.99 (1.37)	2.12 (0.53)	2.45 (1.01)
Serum NfL at baseline (pg/ml)	194.88 (70.8)	142.17 (152.41)	163.25 (126.48)

Continuous variables were presented as means (standard deviations) while categorical variables as absolute numbers (percentages). Disease progression rate at baseline was calculated as the difference between presumed maximal ALSFRS-r score before onset of ALS (31) minus ALSFRS-r score at baseline, divided by time in months from onset to baseline. *data on serum NfL for one male patient is missing.

ALSFRS-r, Amyotrophic Lateral Sclerosis Functional Rating Scale-revised; FVC, forced vital capacity; BMI, body mass index; CRP, C-reactive protein; NLR, neutrophil-to-lymphocytes ratio; NfL, neurofilament light chain.

Nine out of 750 laboratory or anthropometric measurements were imputed (1.2%). Overall, 75 measurements were included in the main analysis, collected in a period starting from 5 months, until 30 months, since the onset of disease. Tregs subpopulations analyses were carried out on a lower number of participants due to missing data. In detail, CD38 and CXCR3 markers were measured for 15 patients with 51 measurements, whereas CD39 and PD1 markers for 16 patients with 55 measurements (**Supplementary Table 2**). Missing values resulted from challenges posed by COVID-19 pandemic and logistical issues in the collection and processing of fresh samples, including limitations in the availability of biological material.

**Figure 2:**
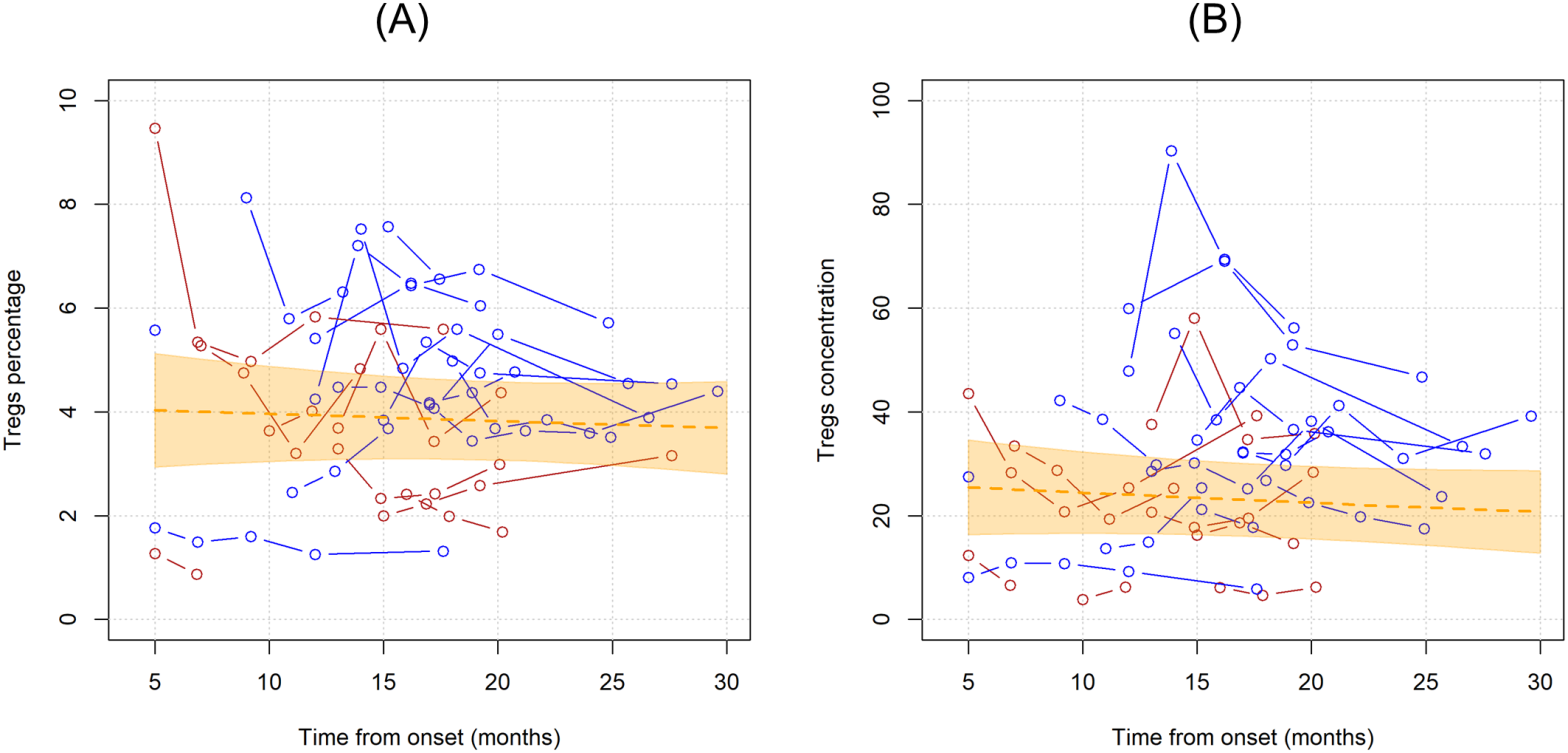
Unadjusted linear trends over time of Tregs percentages over total CD4 T cells **(A)** and Tregs concentrations **(B)**. Continuous lines represent observed trajectories for each single patient with ALS. Female patients’ Tregs trajectories are depicted in red color, while males’ ones in blue. Dotted orange line represents the average linear variation in Tregs percentage **(A)** and concentration **(B)** over time, expressed in months since onset of disease, with 95% confidence interval depicted by the yellow-shaded area. Tregs concentrations are expressed as cell count per microliter.

When considering the time since onset of first motor symptoms of ALS, the unadjusted average monthly variation in Tregs % resulted in a -0.3% decrease (MR=0.997, 95%CI 0.984;1.010, p=0.598). Setting Treg cell count as the dependent variable resulted in a similar non-significant average yearly decrease of -0.8% (MR=0.992, 95%CI 0.975,1.009, p=0.344). Graphical representations of the average trends in Tregs since disease onset with individual variations are provided in **Figure 2**.

### 3.2 Influence of clinical and laboratory variables in Tregs variations in ALS patients

To describe Tregs variations during time, the effect of several demographic, clinical, and laboratory variables was tested by multivariable generalized linear mixed models, in an attempt to account for the clinical heterogeneity of the cohort. **Table 2** shows the mean ratios for each explanatory variable in the multivariable analysis.

**Table 2:** Multivariable model for variations in Tregs, expressed as Tregs percentages (over total CD4 T cells) or Tregs concentrations (cell count/ul).

Variable		Tregs percentage (over total CD4 T cells)			Tregs concentration (cell count/uL)		
		MR	95% CI	p-value	MR	95% CI	p-value
Time from onset	+ 30 days	1.003	0.984; 1.022	0.766	0.997	0.974; 1.022	0.832
ALSFRS-R	- 5 points	0.987	0.913; 1.067	0.738	0.992	0.900; 1.093	0.869
FVC	- 10%	0.976	0.937; 1.016	0.237	0.985	0.935; 1.038	0.573
Site of onset	Bulbar vs Spinal	0.884	0.538; 1.452	0.625	0.630	0.355; 1.121	0.116
BMI	+ 1 kg/m²	0.988	0.905; 1.078	0.783	0.983	0.885; 1.092	0.753
Weight change	- 10 kg	0.865	0.598; 1.250	0.439	0.943	0.601; 1.480	0.799
Age at onset	+ 10 years	0.990	0.835; 1.173	0.906	0.982	0.807; 1.196	0.859
Sex	M vs F	**1.701**	**1.021; 2.832**	**0.041**	1.622	0.897; 2.933	0.110
Albumin	+ 1 g/dL	1.179	0.994; 1.398	0.059	1.175	0.951; 1.452	0.136
CRP	+ 1 (log scale)	1.071	0.972; 1.180	0.164	1.085	0.958; 1.229	0.199
Uric acid	+ 1 mg/dL	0.988	0.904; 1.081	0.799	1.025	0.916; 1.148	0.666
Total cholesterol	+ 100 mg/dL	**1.339**	**1.008; 1.780**	**0.044**	**1.472**	**1.034; 2.095**	**0.032**
Monocytes	+ 0.1 cell/mm³	1.038	0.975; 1.105	0.244	**1.112**	**1.031; 1.200**	**0.006**
Creatinine	+ 1 mg/dL	0.954	0.881; 1.032	0.240	0.944	0.853; 1.045	0.266
Triglycerides	+ 1 (log scale)	0.853	0.695; 1.047	0.129	0.994	0.766; 1.289	0.962
NLR	+ 1 (log scale)	1.219	0.981; 1.515	0.075	**0.725**	**0.557; 0.944**	**0.017**
Serum NfL	+ 1 (log scale)	0.957	0.796; 1.152	0.645	0.964	0.764; 1.216	0.757

Multiplicative effect generalized linear mixed model with gamma distribution and log link function were employed to study Tregs (%) and Treg concentrations variations over time. Mean ratios (MR) with 95% confidence interval (CI) are reported to explain the effect of each variable on Treg % or Treg concentrations, respectively. Significance is set with p-values < 0.05, which are reported in bold character. The mean ratio represents the ratio between average Tregs percentage or concentrations, whichever the dependent variable is. A mean ratio equal to one implies the explanatory variable has an estimated average 0% effect on Tregs; any increase or decrease in the mean ratio over one suggests there is an average percentage increase or decrease in Tregs compared to reference values.

ALSFRS-r, Amyotrophic Lateral Sclerosis Functional Rating Scale-revised; FVC, forced vital capacity; BMI, body mass index; CRP, C-reactive protein; NLR, neutrophil-to-lymphocytes ratio; NFL, neurofilament light chain.

When corrected for all the explanatory variables, time since onset of the disease as well as ALSFRS-r and FVC did not modify Treg percentages or cell counts. Among demographic characteristics, being a male was associated with increased Tregs, especially when considering their percentages over total CD4 T cells (MR = 1.701, 95%CI 1.021;2.832, p= 0.041). Other metabolic markers such as total cholesterol positively correlated with Tregs both when measured as percentages over CD4 T cells and as cell counts, whereas albumin seemed to have positive, though non-significant, association with Tregs(%) (MR=1.179, 95%CI 0.994;1.398, p=0.059). When considering markers of peripheral inflammation, we observed that monocyte counts positively affected Treg cell counts (MR=1.112, 95%CI 1.031;1.200, p=0.006) but not percentages, while NLR negatively correlated with Tregs cell count: for each one-unit increase in the log of this ratio, Treg concentrations decreased on average of 27.5% (MR=0.725, 95%CI 0.557;0.944, p= 0.017). Neither neurofilaments nor CRP modified Tregs.

### 3.3 Effect of Tregs variations over ALSFRS-R and FVC during ALS course

**Figure 3:**
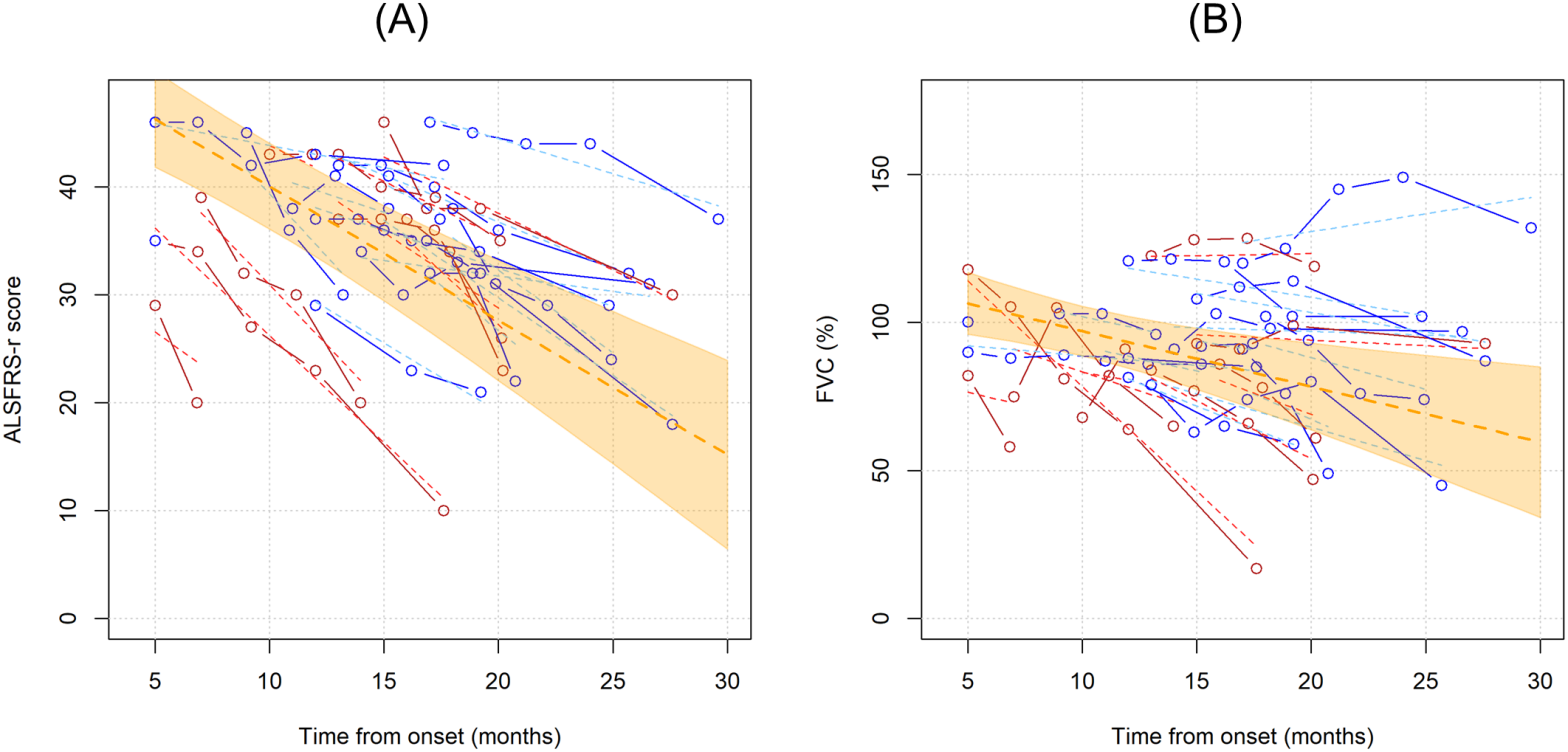
Unadjusted linear trends over time of ALSFRS-r **(A)** and FVC percentages **(B)**. Continuous lines represent observed trajectories for each single patient with ALS, while dotted lines depict predicted trajectories. Female patients’ Tregs trajectories are depicted in red color, while males’ ones in blue. Dotted orange line represents the average linear variation in ALSFRS-r **(A)** and FVC (%) **(B)** over time, expressed in months since onset of disease, with 95% confidence interval depicted by the yellow-shaded area. Predicted values were calculated from an additive effect multivariable linear mixed model which considers clinical and laboratory characteristics as the independent variables.

Next, we wanted to investigate whether Treg number, expressed as percentages over total CD4 T cells or as concentrations, could influence ALSFRS-r or FVC. In the unadjusted analysis, ALSFRS-R declined on average of 1.244 points per month (95%CI -1.636;-0.851, p<0.001) while FVC of 1.873% per month (95%CI -3.078;-0.668, p=0.002) (**Figure 3**). Since the site and age of onset, BMI, weight loss, and neurofilaments are all known prognostic factors for ALS, they were all included as explanatory variables in the models; sex was also included as a covariate. Two separate models including Treg expressed as percentages over total CD4 T cells and as concentrations were run, to investigate which unit of measure better reflects ALSFRS-r or FVC decline. **Table 3** shows the results of the four multivariable mixed models.

**Table 3:** Multivariable model for variations in ALSFRS-r and FVC.

ALSFRS-r							
Variable		Tregs percentage*			Tregs concentration*		
		MD	95% CI	p-value	MD	95% CI	p-value
Time from onset	+ 30 days	**-0.900**	**-1.288; -0.513**	**<0.001**	**-0.928**	**-1.357; -0.499**	**<0.001**
FVC	-10%	**-1.186**	**-1.939; -0.433**	**0.002**	**-1.204**	**-1.971; -0.437**	**0.002**
Tregs	+1 unit *	0.114	-0.662; 0.890	0.773	-0.026	-0.112; 0.060	0.553
Site of onset	Bulbar vs Spinal	2.364	-5.433; 10.160	0.552	1.926	-5.722; 9.573	0.622
BMI	+ 1 kg/m²	-0.947	-2.419; 0.525	0.207	-1.062	-2.522; 0.398	0.154
Weight change	- 10 kg	-5.026	-11.370; 1.318	0.121	-5.543	-11.969; 0.883	0.091
Age at onset	+ 10 years	1.429	-1.275; 4.133	0.300	1.472	-1.153; 4.096	0.272
Sex	M vs F	3.622	-4.075; 11.318	0.356	4.126	-3.284; 11.535	0.275
Serum NfL	+ 1 (log scale)	-0.361	-2.952; 2.229	0.785	-0.521	-3.043; 2.001	0.686
FVC (%)							
Variable		Tregs percentage*			Tregs concentration*		
		MD	95% CI	p-value	MD	95% CI	p-value
Time from onset	+ 30 days	-0.366	-1.636; 0.904	0.573	-0.294	-1.569; 0.982	0.652
ALSFRS-r	- 5 points	**-6.809**	**-10.428; -3.190**	**<0.001**	**-7.108**	**-10.574; -3.642**	**<0.001**
Tregs	+1 unit *	0.929	-1.728; 3.585	0.493	0.291	-0.019; 0.601	0.066
Site of onset	Bulbar vs Spinal	-6.136	-23.533; 11.260	0.489	-3.323	-18.708; 12.061	0.672
BMI	+ 1 kg/m²	1.651	-1.566; 4.869	0.315	1.411	-1.630; 4.453	0.363
Weight change	- 10 kg	2.753	-14.278; 19.785	0.751	5.041	-12.376; 22.458	0.571
Age at onset	+ 10 years	2.472	-3.660; 8.604	0.429	1.763	-3.433; 6.959	0.506
Sex	M vs F	7.246	-10.176; 24.667	0.415	4.499	-11.010; 20.009	0.570
Serum NfL	+ 1 (log scale)	2.230	-5.666; 10.127	0.580	1.585	-5.635; 8.804	0.667

Additive effect linear mixed models were used to investigate ALSFRS-R and FVC variations over time. Mean differences (MD) with 95% confidence interval (CI) are reported to explain the effect of each variable on ALSFRS-R and FVC. Four models were analyzed. In the upper section of the table, two models were constructed with ALSFRS-R variation over time as the dependent variable, and the independent variables listed in the first column. Among these independent variables, Tregs were included as percentages in the upper left portion of the table and as concentrations in the upper right portion.

In the lower section of the table, two additional models were constructed with FVC% variation over time as the dependent variable, and the independent variables listed in the first column. Similarly, Tregs were included as percentages in the lower left portion of the table and as concentrations in the lower right portion.

Significance is set with p-values < 0.05, which are reported in bold characters. A positive mean difference implies that the variation in the explanatory variable is associated to an increase in ALSFRS-r or FVC scores, whichever the dependent variable is, whereas a negative sign in the mean difference suggests that the variation in the explanatory variable is associated to a decrease in ALSFRS-r or FVC scores.

*for both ALSFRS-r and FVC, the effect of Tregs in the first model is related to a 1% increase, whereas in the second model to a 1 cell count/uL increase.

ALSFRS-r, Amyotrophic Lateral Sclerosis Functional Rating Scale-revised; FVC, forced vital capacity; BMI, body mass index; NFL, neurofilament light chain.

FVC and ALSFRS-r decline were tightly connected: for each 10% decrease in FVC, ALSFRS-r score declines on average by 1.204 points (95%CI -1.971;-0.4374, p= 0.002, when Treg concentrations were considered in the model, see upper right portion of the **Table 3**), and vice versa, every 5 points drop in ALSFRS-r corresponds to an average decline of 7.1081% in FVC (95%CI -10.574;-3.642, p<=0.0001, see lower right portion of the **Table 3**), independently of the decline due to the time course of the disease. Similar results were observed when Tregs percentages instead of concentrations were included in the model (left portion of **Table 3**).

Notably, Treg percentage variations did not influence either ALSFRS-r or FVC decline, while Treg cell counts had a positive, though non-significant effect, on FVC scores: every single Treg cell count decrease over one microliter corresponded to an average drop of 0.291% of FVC (95%CI -0.601;0.019, p=0.066). This effect was replicated when running a more simplified model including only time since onset of the disease and Treg variations. Among all the explanatory variables included in the multivariable models, only weight loss slightly affected ALSFRS-r decline, though without reaching statistical significance (every 10 kilograms loss corresponded to an average decline of 5.543 points when Treg concentrations were accounted, 95%CI-11.969;0.883, p= 0.091). All the other explanatory variables, serum NfL included, were not independently associated with ALSFRS-r or FVC declines over time.

### 3.4 Tregs phenotype modifies during ALS course

**Figure 4:**
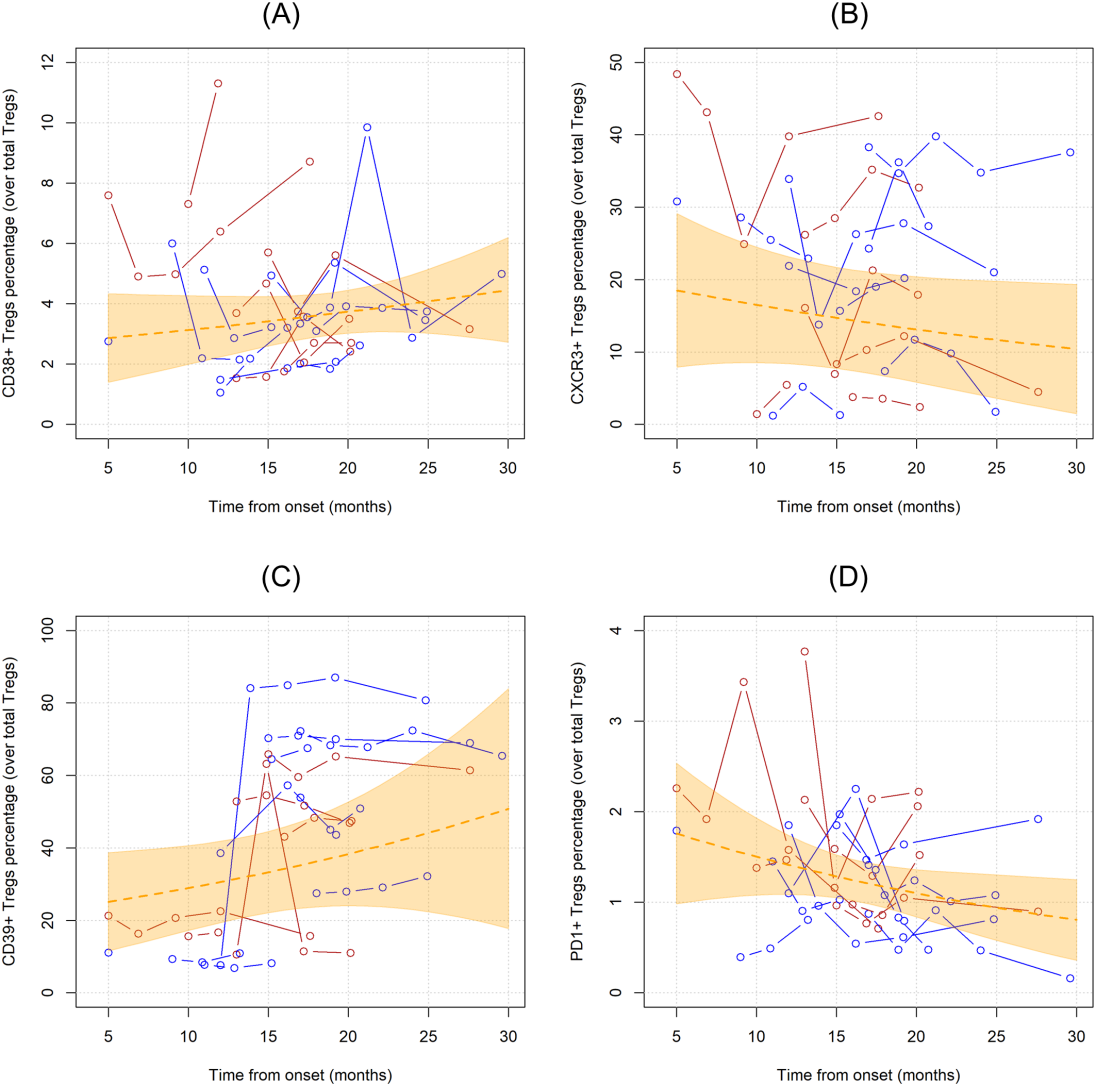
Unadjusted linear trends over time of CD38+ **(A)**, CXCR3+ **(B)**, CD39+ **(C)**, and PD1+ **(D)** Tregs subpopulations expressed in percentages over total Tregs. Continuous lines represent observed trajectories for each single patient with ALS. Female patients’ Tregs trajectories are depicted in red color, while males’ ones in blue. Dotted orange line represents the average linear variation in CD38+ Tregs **(A)**, CXCR3+ Tregs **(B)**, CD39+ Tregs **(C)**, and PD1+ Tregs **(D)** over time, expressed in months since onset of disease, with 95% confidence interval depicted by the yellow-shaded area. Tregs subpopulations are expressed as the percentage over total Tregs.

CD38, CXCR3, CD39, PD1 markers were employed to characterize Tregs. In the first analysis, the unadjusted linear trends of these subpopulations were investigated (**Figure 4**). Notably, PD1+ Tregs significantly declined over time when considered as percentages over total CD4 T cells (MR= 0.956, 95%CI 0.921;0.993, p=0.019), while when measured as percentages over Tregs and as concentrations this trend did not reach statistical significance (**Supplementary Table 2**). CD39+Tregs, when measured as percentages over total Tregs, showed a non-significant linear increase over time (MR=1.029, 95%CI 0.989;1.070, p=0.159).

Next, we run multivariable models including all the abovementioned variables, whose results are summarized in **Table 4** and **Supplementary Tables 3**-**6**.

**Table 4:** Multivariable model for variations in Tregs subpopulations defined by expression of markers CD38, CD39, CXCR3, PD1, expressed as percentages over total Tregs.

Variable		CD38+ Tregs			CXCR3+ Tregs		
		MR	95% CI	p-value	MR	95% CI	p-value
Time from onset	+ 30 days	1.023	0.986; 1.062	0.220	1.024	0.960; 1.092	0.476
ALSFRS-R	- 5 points	0.868	0.712; 1.059	0.163	0.815	0.603; 1.101	0.183
FVC	- 10%	**1.142**	**1.031; 1.264**	**0.011**	1.046	0.899; 1.218	0.559
Site of onset	Bulbar vs Spinal	0.941	0.651; 1.360	0.747	0.624	0.213; 1.828	0.390
BMI	+ 1 kg/m²	0.961	0.843; 1.097	0.559	0.896	0.651; 1.235	0.503
Weight change	- 10 kg	0.739	0.381; 1.434	0.371	0.785	0.176; 3.501	0.751
Age at onset	+ 10 years	1.113	0.958; 1.293	0.163	0.797	0.539; 1.179	0.256
Sex	M vs F	1.004	0.569; 1.772	0.989	1.919	0.493; 7.474	0.348
Albumin	+ 1 g/dL	1.512	0.730; 3.132	0.265	**0.286**	**0.106; 0.773**	**0.014**
CRP	+ 1 (log scale)	**1.330**	**1.033; 1.712**	**0.027**	1.275	0.809; 2.012	0.296
Uric acid	+ 1 mg/dL	1.007	0.837; 1.212	0.938	1.306	0.890; 1.915	0.173
Total cholesterol	+ 100 mg/dL	**1.968**	**1.203; 3.221**	**0.007**	**3.058**	**1.226; 7.626**	**0.017**
Monocytes	+ 0.1 mm³	0.969	0.877; 1.071	0.543	0.995	0.838; 1.183	0.957
Creatinine	+ 1 mg/dL	1.078	0.956; 1.216	0.220	0.888	0.656; 1.203	0.444
Triglycerides	+ 1 (log scale)	0.720	0.430; 1.203	0.210	0.876	0.427; 1.796	0.718
NLR	+ 1 (log scale)	1.098	0.722; 1.668	0.662	1.075	0.596; 1.937	0.811
NFL in serum	+ 1 (log scale)	**0.696**	**0.515; 0.940**	**0.018**	0.891	0.539; 1.473	0.653
Variable		CD39+ Tregs			PD1+ Tregs		
		MR	95% CI	p-value	MR	95% CI	p-value
Time from onset	+ 30 days	**1.112**	**1.046; 1.183**	**0.001**	**0.939**	**0.894; 0.987**	**0.013**
ALSFRS-R	- 5 points	**0.707**	**0.552; 0.907**	**0.006**	1.219	0.987; 1.506	0.067
FVC	- 10%	1.055	0.926; 1.201	0.423	0.960	0.860; 1.072	0.468
Site of onset	Bulbar vs Spinal	0.589	0.330; 1.050	0.073	0.771	0.322; 1.851	0.561
BMI	+ 1 kg/m²	0.953	0.783; 1.159	0.627	1.075	0.833; 1.386	0.580
Weight change	- 10 kg	0.480	0.200; 1.151	0.100	1.233	0.419; 3.628	0.703
Age at onset	+ 10 years	**1.251**	**1.026; 1.526**	**0.027**	1.164	0.855; 1.583	0.335
Sex	M vs F	0.996	0.440; 2.252	0.992	0.632	0.206; 1.942	0.423
Albumin	+ 1 g/dL	0.778	0.338; 1.791	0.556	1.214	0.583; 2.529	0.604
CRP	+ 1 (log scale)	1.098	0.785; 1.534	0.585	1.186	0.837; 1.681	0.337
Uric acid	+ 1 mg/dL	1.191	0.831; 1.705	0.341	0.880	0.692; 1.120	0.299
Total cholesterol	+ 100 mg/dL	0.878	0.427; 1.808	0.725	0.771	0.388; 1.532	0.458
Monocytes	+ 0.1 mm³	1.040	0.910; 1.188	0.568	0.881	0.768; 1.011	0.072
Creatinine	+ 1 mg/dL	0.954	0.811; 1.122	0.568	1.189	0.969; 1.459	0.098
Triglycerides	+ 1 (log scale)	1.810	0.897; 3.654	0.098	**1.789**	**1.066; 3.001**	**0.028**
NLR	+ 1 (log scale)	1.242	0.742; 2.078	0.410	1.230	0.788; 1.920	0.362
NFL in serum	+ 1 (log scale)	**1.548**	**1.000; 2.395**	**0.050**	1.372	0.920; 2.045	0.121

Multiplicative effect generalized linear mixed model with gamma distribution and log link function were employed to study each Treg subpopulation variation. Mean ratios (MR) with 95% confidence interval (CI) are reported to explain the effect of each variable on Treg % or Treg concentrations, respectively. Significance is set with p-values < 0.05, which are reported in bold character. The mean ratio represents the ratio between each subpopulation’s average Tregs percentage.

ALSFRS-r, Amyotrophic Lateral Sclerosis Functional Rating Scale-revised; FVC, forced vital capacity; BMI, body mass index; CRP, C-reactive protein; NLR, neutrophil-to-lymphocytes ratio; NFL, neurofilament light chain.

When considering each subpopulation in percentages over total Tregs (**Table 4**), an opposite trend could be observed between CD39+ and PD1+Tregs, with the former increasing every month since onset of the disease by an average 11% (MR= 1.112, 95%CI 1.046;1.183, p=0.001) and the latter decreasing on average by 6% (MR= 0.939, 95%CI 0.894;0.987, p=0.013). Similarly, the decline in ALSFRS-r resulted in opposite effects on these two subpopulations still when assuming time since onset of the disease, with CD39+Tregs decreasing for each 5-point loss in ALSFRS-r while PD1+ increased, though without reaching statistical significance (MR= 1.219, 95% CI 0.987;1.506, p=0.067).

Moreover, in the adjusted multivariable models, CD38+Treg counts showed a significant increase over time (accounted as months since onset of the disease) (**Supplementary Table 3**). Regarding other clinical variables associated with ALS, weight loss was an independent factor in decreasing CD38+Treg concentrations (MR=0.404, 95%CI 0.188-0.869,p=0.020), while age at onset influenced CD39+Tregs(%) over total Tregs (MR=1.251,95%CI 1.026;1.526, p=0.027). Total cholesterol species determined a rise over time of CD38+ and CXCR3+Tregs (**Supplementary Tables 3**, **5**), while triglycerides acted more on PD1+Tregs, by opposing their longitudinal trend towards a decrease over time. Albumin affected CXCR3+Tregs by decreasing their levels in every unit of measure they were considered.

Finally, by incorporating serum NfL in the multivariable model, we could observe an opposite effect of this marker of neurodegeneration on Treg subpopulations: while decreasing CD38+Tregs (MR= 0.696, 95%CI 0.515;0.940, p=0.018 for CD38+Tregs/total Tregs), each log unit increase in serum NfL increased PD1+Tregs (MR=1.495, 95%CI 1.010-2.213, p=0.044 for PD1+Tregs/total CD4) and CD39+Treg, though the latter with minimal statistical significance when considered as CD39+Tregs/total Tregs (MR=1.548, 95%CI 1.000-2.395, p=0.050).

## 4 Discussion

In this explorative longitudinal study, we suggest that Tregs number does not change over time in ALS patients; lipid and in particular cholesterol metabolism, peripheral inflammation, and gender might impact Treg fluctuations during the natural history of ALS and should be considered when designing and interpreting clinical trials in which Treg levels are used as biomarkers. The characterization of Tregs phenotype by analysis of the four surface markers (CD38, CXCR3, CD39, PD1) was limited to the percentage of Tregs expressing each marker at the time, without considering distinct or overlapping subpopulations. However, when phenotyping within the Treg population, this preliminary analysis showed PD1+Tregs might change during ALS course with a decrease over time, while CD39+Tregs may tend to expand, especially when ALSFRS-r score does not vary over time. PD1+ Tregs decreasing over time since onset of the disease might suggest a progressive decline in their immunoregulatory capacity, potentially contributing to the heightened inflammation observed in ALS. On the other hand, the expansion of CD39+ Tregs could represent a compensatory mechanism attempting to counteract neuroinflammation through enhanced adenosine production, which has anti-inflammatory effects. However, given the small sample size in our study and lack of supportive functional studies, the observed trend require confirmation in larger cohorts before definitive conclusions can be drawn about their role in ALS pathophysiology.

This study also meant to attempt to explain part of the intricate relationship between Tregs and other variables as sex, age, cholesterol, and inflammation, during ALS clinical course. The advantage of such an approach is to synthesize all variables at once considering their variations in time; however, we recognize these findings merely “scratch the surface” of a much more complicated and dynamic balance between biological phenomena and the validation of the relation of each explanatory variable with Tregs would require a whole set of functional experiments per se. Besides, the incorporation of all these explanatory variables in a multivariable model could be subject to statistical bias given the limited sample size of our study, and the lack of a validation cohort; however, the results of the regression coefficients for each variable were fairly consistent across all the multivariable models run for every dependent variable when measured in different units of measure.

With these limits in mind, the association between Tregs and cholesterol metabolism has been already described in genetic hyperlipidemias, where excess circulating cholesterol species are sensed by Tregs and impair their function and proliferative capacities (32). In the general population, HDL was found to positively associated with Tregs (33), and rosuvastatin administration decreased the number of peripheral Tregs in normocholesterolemic subjects (34). In familial hypercholesterolemia patients, increased Treg frequencies were found, though their suppressive capacities were impaired and impacted atherosclerosis progression (35). On the other hand, recent trials on autologous Tregs transplantation in ALS patients indicate oxidized-LDL species decreased following this intervention (36), suggesting that functional Tregs may act on the reverse on circulating lipid species attenuating overall oxidative stress mediated by them. In our study, the positive association between total cholesterol and Tregs was particularly evident for CD38+ and CXCR3+ subsets, probably reflecting a switch to a more metabolically active profile with higher homing capacities.

When looking at all the explanatory variables for Tregs variations concerning inflammation, the abovementioned inherent limits of our observational work may hold, along with the risk of reverse causation, and the necessity of further functional experiments to clarify the sequential regulation of each immunological player during the ALS clinical course.

NLR, a known marker of all-cause mortality in the general population as well as in ALS patients (25, 27) in our study seemed to modify Tregs. Not surprisingly, CRP, a known marker of systemic inflammation, predicted higher levels of metabolically activated CD38+ and PD1+Tregs without affecting total Treg concentrations or percentages over total CD4 T cells. Besides, in our analysis, we could observe a positive association between Tregs and monocytes in multivariable analysis, suggesting an independent role of this variable. Interestingly, monocytes were proven to longitudinally increase over time in ALS patients (37). Other works have explored the role of activated monocytes in driving ALS progression (26, 28, 38, 39).

Males seem to have increased Tregs, especially metabolically active CD38-positive, during the ALS course. This is in line with previous works in which Treg frequencies were found to be increased in healthy male subjects (40, 41). Sex-specific differences in ALS prognosis dictated by peripheral blood cell counts have been recently uncovered spanning several immune cells, from neutrophils to natural killer cells (25, 42), raising the possibility of an interplay between sexual hormones and immune regulation in ALS.

More recently, the balance between activated and resting Tregs in contrast with the proportion of T effector cells have been described to have opposite effects on survival, with protective or noxious consequences respectively (43). PD1 surface expression within T cells could be associated to exhaustion only when co-expression of other markers and *in vivo* tests of cytokine production are performed, otherwise the effects downstream this receptor-ligand interaction could be so multifaced it would be reductive to tag as “exhausting” Tregs those expressing PD1 (44). In previous studies on multiple sclerosis, PD1+Tregs were found to be altered compared to healthy controls (45), and disease-modifying treatments could enhance the surface expression of PD1 on Tregs (46). In our study, though we limited our analysis to the surface expression of this marker, PD1+Tregs seemed to decrease with ALS course since the onset of the disease with an inverse correlation with ALSFRS-r decline. Conversely, CD39-expressing Tregs identify a subpopulation of Tregs with enhanced suppressive capacities due to the ATPase activity of this membrane protein (47) and that was found to be increased over time since the start of symptoms in our ALS cohort, especially when the ALSFRS-r score did not decrease as expected. These data may suggest that, though total Tregs do not longitudinally modify because of ALS clinical course, their phenotype might change. Intriguingly, serum NfL, which could reflect the neurodegenerative pace, affects only certain markers-expressing Tregs, in particular CD38+Tregs, which decreased for each increase in serum NfL, and CD39+ or PD1+Treg, which on the other hand, expanded upon NfL increase.

In the authors’ opinion, the prognostic implications of such observations deserve further research and could be interesting markers for monitoring the disease progression.

The relation between Treg levels with ALSFRS-r decline was not supported by our study. In this setting, however, FVC could be more sensitive than ALSFRS-R to Treg concentrations increase, and the instrumental monitoring of respiratory function in ALS efficiently predicts the disease evolution towards support procedures (48). Longitudinal models of ALSFRS-r or FVC decline in our cohort confirmed the strong relation between these two clinical measures of ALS one to the other, independently of time, as already reported in other studies (31). The other clinical and biological variables did not seem to account for variations in ALSFRS-r and FVC, highlighting that the decline in these measures may be mainly related to time and not to other individual characteristics.

The importance of including several clinical and demographic variables besides time in regression models for explaining the variations of Tregs numbers during the ALS course is paramount to understanding how intricate the relation between T regulatory cells and immune homeostasis in patients is. Limiting our observations for time wouldn’t have allowed us to predict Tregs fluctuations and, above all, would have misled us to the hypothesis Tregs number does not vary over time in ALS and might be used as an efficient pharmacodynamic marker for ALS. Our analysis is limited by the analysis of a single cohort, with restricted sample size and number of observations; besides, due to the small number of observations per subject, it was not possible to include several random slopes in mixed models to account for individual variations of each independent variable’s effect on the outcomes. Moreover, the findings of our study are limited to the ALS course period ranging from about 5 to 30 months from disease onset.

Notwithstanding these inherent limitations, our findings should prompt further longitudinal studies in larger and multiple cohorts of ALS patients, collecting more Tregs assessments over time per subject, to validate these results.

## 5 Conclusions

In conclusion, though Treg number appears to be constant over the disease course and does not affect ALSFRS-r decline, our study suggests that Tregs widely fluctuate and may differently vary in response to contingencies, such as inflammatory or metabolic (especially lipidic) triggers, or in a sex-specific manner, though these results should be confirmed in other cohorts and larger studies. This should be considered and possibly discourages choosing Treg levels as pharmaco-dynamic markers for ALS clinical trial data interpretation.

Furthermore, the characterization of Treg phenotypic activity through the analysis of surface marker expression has offered the opportunity to better explain some of the variations in Tregs during the course of ALS since the onset of symptoms and has shed light on PD1+ and CD39+Tregs, which tend to vary and positively correlate with markers of neurodegeneration. This preliminary insight on how Tregs might vary in phenotype with disease progression should be the subject of broader functional investigations.

## Data Availability

The raw data supporting the conclusions of this article will be made available by the authors to external researchers who provide scientific proposals and whose proposed use of the data has been approved by an independent review committee identified for this purpose.
