# Supplementary material for: Tregs levels and phenotype modifications during Amyotrophic Lateral Sclerosis course
Source: Front Immunol. 2025 Jan 8;15:1508974. doi: 10.3389/fimmu.2024.1508974 (PMC11750661; doi:10.3389/fimmu.2024.1508974)
Supplement: Supplementary file 1 [file Table1.docx]

**Supplementary Materials**

Supplementary table 1…………………………………………………………………………………………………2

Supplementary table 2…………………………………………………………………………………………………3

Supplementary table 3…………………………………………………………………………………………………4

Supplementary table 4…………………………………………………………………………………………………5

Supplementary table 5…………………………………………………………………………………………………6

Supplementary table 6…………………………………………………………………………………………………7

Supplementary table 7…………………………………………………………………………………………………8

**Supplementary Table 1. Descriptive statistics of Tregs and Tregs subpopulations at study baseline.** Tregs were expressed as percentages (over total CD4 T cells) or concentrations (absolute count/ul), whereas CD38+, CXCR3+ CD39+ and PD1+ Tregs subpopulations were expressed as percentages (over total Tregs), percentages (over total CD4 T cells) or concentrations (absolute count/ul). All variables are continuous and were presented as means (standard deviations). *: data for one male patient is missing.

| **Tregs** | **Females**  **(n=8)** | **Males**  **(n=13)** | **Total**  **(n=21)** |
| --- | --- | --- | --- |
| Tregs percentage (over total CD4 T cells) * | 3.88 (2.57) | 4.73 (1.82) | 4.39 (2.13) |
| Tregs concentration (count/uL) * | 21.74 (14.87) | 34.06 (15.36) | 29.13 (16.01) |
| **Tregs subpopulations** | **Females**  **(n=6)** | **Males**  **(n=9)** | **Total**  **(n=15)** |
| CD38+ Tregs percentage (over total Tregs) * | 4.60 (2.68) | 3.11 (1.72) | 3.75 (2.22) |
| CD38+ Tregs concentration (count/uL) * | 0.99 (1.17) | 0.99 (0.64) | 0.99 (0.87) |
| CD38+ Tregs percentage (over total CD4 T cells) * | 0.22 (0.26) | 0.16 (0.14) | 0.18 (0.19) |
| CD39+ Tregs percentage (over total Tregs) | 34.88 (22.35) | 33.14 (26.81) | 33.84 (24.28) |
| CD39+ Tregs concentration (count/uL) | 6.36 (4.49) | 11.91 (9.92) | 9.69 (8.45) |
| CD39+ Tregs percentage (over total CD4 T cells) | 1.18 (0.64) | 1.48 (1.06) | 1.36 (0.90) |
| CXCR3+ Tregs percentage (over total Tregs) * | 17.38 (17.67) | 23.29 (12.92) | 20.76 (14.80) |
| CXCR3+ Tregs concentration (count/uL) * | 5.98 (8.23) | 9.03 (5.60) | 7.72 (6.74) |
| CXCR3+ Tregs percentage (over total CD4 T cells) * | 1.06 (1.76) | 1.21 (0.74) | 1.15 (1.22) |
| PD1+ Tregs percentage (over total Tregs) | 1.91 (1.07) | 1.31 (0.50) | 1.55 (0.80) |
| PD1+ Tregs concentration (count/uL) | 0.52 (0.56) | 0.45 (0.24) | 0.48 (0.38) |
| PD1+ Tregs percentage (over total CD4 T cells) | 0.09 (0.08) | 0.06 (0.02) | 0.07 (0.05) |

**Supplementary Table 2. Unadjusted monthly variation of Tregs and Tregs subpopulations.** Multiplicative effect generalized linear mixed model with gamma distribution and log link function were employed to study Tregs (%) and Treg concentrations unadjusted variations over time. Mean ratios (MR) with 95% confidence interval (CI) are reported to explain the average observed monthly variation of Treg % or Treg concentrations, respectively. Significance is set with p-values < 0.05, which are reported in bold character. The mean ratio represents the ratio between average Tregs percentage or concentrations, whichever the dependent variable is. A mean ratio equal to one implies that a one-months increase in time has an estimated average 0% effect on Tregs; any increase or decrease in the mean ratio over one suggests there is an average monthly increase or decrease in Tregs.

| **Tregs** | **MR** | **95% CI** | **p** |
| --- | --- | --- | --- |
| Tregs percentage (over total CD4 T cells) | 0.997 | 0.984 ; 1.010 | 0.598 |
| Tregs concentration (count/uL) | 0.992 | 0.975 ; 1.009 | 0.344 |
| CD38+ Tregs percentage (over total Tregs) | 1.018 | 0.985 ; 1.052 | 0.287 |
| CD38+ Tregs concentration (count/uL) | 1.008 | 0.959 ; 1.059 | 0.766 |
| CD38+ Tregs percentage (over total CD4 T cells) | 1.012 | 0.970 ; 1.055 | 0.590 |
| CD39+ Tregs percentage (over total Tregs) | 1.029 | 0.989 ; 1.070 | 0.159 |
| CD39+ Tregs concentration (count/uL) | 1.019 | 0.969 ; 1.072 | 0.466 |
| CD39+ Tregs percentage (over total CD4 T cells) | 1.014 | 0.968 ; 1.063 | 0.546 |
| CXCR3+ Tregs percentage (over total Tregs) | 0.977 | 0.937 ; 1.019 | 0.284 |
| CXCR3+ Tregs concentration (count/uL) | 0.998 | 0.942 ; 1.057 | 0.946 |
| CXCR3+ Tregs percentage (over total CD4 T cells) | 0.989 | 0.951 ; 1.028 | 0.577 |
| PD1+ Tregs percentage (over total Tregs) | 0.969 | 0.934 ; 1.006 | 0.098 |
| PD1+ Tregs concentration (count/uL) | 0.967 | 0.926 ; 1.010 | 0.129 |
| PD1+ Tregs percentage (over total CD4 T cells) | **0.956** | **0.921 ; 0.993** | **0.019** |

**Supplementary Table 3. Multivariable model for variations in CD38+ Tregs, expressed as percentages (over total Tregs) or concentrations (absolute count/ul).** Multiplicative effect generalized linear mixed model with gamma distribution and log link function were employed to study CD38+ Tregs (% over total CD4 T cells) and CD38+ Tregs concentrations variations over time. Mean ratios (MR) with 95% confidence interval (CI) are reported to explain the effect of each variable on CD38+ Treg % or CD38+ Treg concentrations, respectively. Significance is set with p-values < 0.05, which are reported in bold character. The mean ratio represents the ratio between average Tregs percentage or concentrations, whichever the dependent variable is. A mean ratio equal to one implies the explanatory variable has an estimated average 0% effect on CD38+ Tregs; any increase or decrease in the mean ratio over one suggests there is an average percentage increase or decrease in CD38+ Tregs compared to reference values.

| **Variable** | | **CD38+ Tregs percentage**  **(over total CD4 T cells)** | | | **CD38+ Tregs concentration**  **(count/uL)** | | |
| --- | --- | --- | --- | --- | --- | --- | --- |
|  |  | **MR** | **95% CI** | **p-value** | **MR** | **95% CI** | **p-value** |
| Time from onset | + 30 days | 1.031 | 0.989 ; 1.074 | 0.152 | **1.053** | **1.008 ; 1.099** | **0.020** |
| ALSFRS-R | - 5 points | 0.906 | 0.730 ; 1.124 | 0.369 | 0.837 | 0.671 ; 1.045 | 0.117 |
| FVC | - 10 % | **1.132** | **1.015 ; 1.262** | **0.026** | 1.119 | 0.998 ; 1.255 | 0.054 |
| Site of onset | Bulbar vs Spinal | 0.896 | 0.608 ; 1.321 | 0.579 | 0.819 | 0.529 ; 1.268 | 0.370 |
| BMI | + 1 kg/m² | 0.962 | 0.832 ; 1.112 | 0.601 | 0.918 | 0.790 ; 1.067 | 0.267 |
| Weight change | - 10 kg | 0.515 | 0.244 ; 1.086 | 0.081 | **0.404** | **0.188 ; 0.869** | **0.020** |
| Age at onset | + 10 years | 1.018 | 0.868 ; 1.193 | 0.830 | 0.927 | 0.785 ; 1.094 | 0.370 |
| Sex | M vs F | 1.919 | 1.037 ; 3.551 | 0.038 | **2.181** | **1.140 ; 4.172** | **0.018** |
| Albumin | + 1 g/dL | 1.230 | 0.577 ; 2.625 | 0.592 | 0.943 | 0.435 ; 2.048 | 0.883 |
| CRP | + 1 (log scale) | **1.558** | **1.190 ; 2.040** | **0.001** | **1.607** | **1.196 ; 2.160** | **0.002** |
| Uric acid | + 1 mg/dL | 1.066 | 0.864 ; 1.316 | 0.550 | 1.144 | 0.925 ; 1.416 | 0.214 |
| Total cholesterol | + 100 mg/dL | **4.134** | **2.470 ; 6.918** | **0.000** | **5.254** | **3.040 ; 9.081** | **0.000** |
| Monocytes | + 0.1 mm³ | 0.969 | 0.870 ; 1.079 | 0.567 | 1.100 | 0.979 ; 1.235 | 0.109 |
| Creatinine | + 1 mg/dL | 1.086 | 0.955 ; 1.236 | 0.210 | 0.917 | 0.787 ; 1.067 | 0.262 |
| Triglycerides | + 1 (log scale) | **0.492** | **0.278 ; 0.870** | **0.015** | 0.609 | 0.298 ; 1.243 | 0.173 |
| NLR | + 1 (log scale) | **1.643** | **1.059 ; 2.548** | **0.027** | 0.908 | 0.574 ; 1.437 | 0.680 |
| Serum NfL | + 1 (log scale) | **0.721** | **0.528 ; 0.984** | **0.039** | 0.807 | 0.583 ; 1.115 | 0.194 |

Abbreviations: ALSFRS-r: Amyotrophic Lateral Sclerosis Functional Rating Scale-revised; FVC: forced vital capacity; BMI: body mass index; CRP: C-reactive protein; NLR: neutrophil-to-lymphocytes ratio; NfL: neurofilament light chain.

**Supplementary Table 4. Multivariable model for variations in CD39+ Tregs, expressed as percentages (over total Tregs) or concentrations (absolute count/ul).** Multiplicative effect generalized linear mixed model with gamma distribution and log link function were employed to study CD39+ Tregs (% over total CD4 T cells) and CD39+ Tregs concentrations variations over time. Mean ratios (MR) with 95% confidence interval (CI) are reported to explain the effect of each variable on CD39+ Treg % or CD39+ Treg concentrations, respectively. Significance is set with p-values < 0.05, which are reported in bold character. The mean ratio represents the ratio between average Tregs percentage or concentrations, whichever the dependent variable is. A mean ratio equal to one implies the explanatory variable has an estimated average 0% effect on CD39+ Tregs; any increase or decrease in the mean ratio over one suggests there is an average percentage increase or decrease in CD39+ Tregs compared to reference values.

| **Variable** | | **CD39+ Tregs percentage**  **(over total CD4 T cells)** | | | **CD39+ Tregs concentration**  **(count/uL)** | | |
| --- | --- | --- | --- | --- | --- | --- | --- |
|  |  | **MR** | **95% CI** | **p-value** | **MR** | **95% CI** | **p-value** |
| Time from onset | + 30 days | **1.104** | **1.033 ; 1.180** | **0.004** | **1.112** | **1.025 ; 1.208** | **0.011** |
| ALSFRS-R | - 5 points | **0.695** | **0.518 ; 0.932** | **0.015** | **0.673** | **0.488 ; 0.929** | **0.016** |
| FVC | - 10 % | 1.065 | 0.906 ; 1.252 | 0.448 | 1.065 | 0.897 ; 1.263 | 0.474 |
| Site of onset | Bulbar vs Spinal | 0.557 | 0.274 ; 1.129 | 0.105 | 0.429 | 0.172 ; 1.067 | 0.069 |
| BMI | + 1 kg/m² | 0.984 | 0.774 ; 1.250 | 0.893 | 0.959 | 0.713 ; 1.290 | 0.784 |
| Weight change | - 10 kg | 0.440 | 0.141 ; 1.372 | 0.157 | 0.395 | 0.100 ; 1.565 | 0.186 |
| Age at onset | + 10 years | 1.205 | 0.933 ; 1.557 | 0.154 | 1.168 | 0.842 ; 1.621 | 0.352 |
| Sex | M vs F | 1.749 | 0.630 ; 4.855 | 0.283 | 2.054 | 0.586 ; 7.201 | 0.261 |
| Albumin | + 1 g/dL | 0.637 | 0.235 ; 1.725 | 0.375 | 0.519 | 0.174 ; 1.548 | 0.239 |
| CRP | + 1 (log scale) | 1.266 | 0.840 ; 1.907 | 0.260 | 1.241 | 0.790 ; 1.951 | 0.348 |
| Uric acid | + 1 mg/dL | 1.248 | 0.786 ; 1.981 | 0.348 | 1.245 | 0.791 ; 1.960 | 0.344 |
| Total cholesterol | + 100 mg/dL | 1.452 | 0.593 ; 3.559 | 0.415 | 1.601 | 0.586 ; 4.370 | 0.359 |
| Monocytes | + 0.1 mm³ | 1.050 | 0.897 ; 1.230 | 0.542 | 1.174 | 0.985 ; 1.399 | 0.074 |
| Creatinine | + 1 mg/dL | 0.944 | 0.770 ; 1.158 | 0.582 | 0.870 | 0.678 ; 1.117 | 0.275 |
| Triglycerides | + 1 (log scale) | 1.617 | 0.696 ; 3.760 | 0.264 | 2.027 | 0.828 ; 4.964 | 0.122 |
| NLR | + 1 (log scale) | 1.715 | 0.958 ; 3.069 | 0.069 | 1.018 | 0.532 ; 1.948 | 0.957 |
| Serum NfL | + 1 (log scale) | 1.647 | 0.982 ; 2.762 | 0.059 | 1.648 | 0.843 ; 3.220 | 0.144 |

Abbreviations: ALSFRS-r: Amyotrophic Lateral Sclerosis Functional Rating Scale-revised; FVC: forced vital capacity; BMI: body mass index; CRP: C-reactive protein; NLR: neutrophil-to-lymphocytes ratio; NfL: neurofilament light chain.

**Supplementary Table 5. Multivariable model for variations in CXCR3+ Tregs, expressed as percentages (over total Tregs) or concentrations (absolute count/ul).** Multiplicative effect generalized linear mixed model with gamma distribution and log link function were employed to study CXCR3+ Tregs (% over total CD4 T cells) and CXCR3+ Tregs concentrations variations over time. Mean ratios (MR) with 95% confidence interval (CI) are reported to explain the effect of each variable on CXCR3+ Treg % or CXCR3+ Treg concentrations, respectively. Significance is set with p-values < 0.05, which are reported in bold character. The mean ratio represents the ratio between average Tregs percentage or concentrations, whichever the dependent variable is. A mean ratio equal to one implies the explanatory variable has an estimated average 0% effect on CXCR3+ Tregs; any increase or decrease in the mean ratio over one suggests there is an average percentage increase or decrease in CXCR3+ Tregs compared to reference values.

| **Variable** | | **CXCR3+ Tregs percentage**  **(over total CD4 T cells)** | | | **CXCR3+ Tregs concentration**  **(count/uL)** | | |
| --- | --- | --- | --- | --- | --- | --- | --- |
|  |  | **MR** | **95% CI** | **p-value** | **MR** | **95% CI** | **p-value** |
| Time from onset | + 30 days | 1.057 | 0.977 ; 1.143 | 0.169 | 1.050 | 0.967 ; 1.139 | 0.248 |
| ALSFRS-R | - 5 points | 0.761 | 0.532 ; 1.088 | 0.135 | 0.743 | 0.516 ; 1.069 | 0.110 |
| FVC | - 10 % | 1.069 | 0.905 ; 1.261 | 0.433 | 1.055 | 0.883 ; 1.260 | 0.554 |
| Site of onset | Bulbar vs Spinal | 0.554 | 0.132 ; 2.333 | 0.421 | 0.419 | 0.088 ; 2.002 | 0.276 |
| BMI | + 1 kg/m² | 0.875 | 0.582 ; 1.315 | 0.520 | 0.888 | 0.575 ; 1.372 | 0.594 |
| Weight change | - 10 kg | 0.476 | 0.078 ; 2.915 | 0.422 | 0.621 | 0.087 ; 4.405 | 0.633 |
| Age at onset | + 10 years | 0.772 | 0.457 ; 1.305 | 0.334 | 0.728 | 0.415 ; 1.277 | 0.269 |
| Sex | M vs F | 3.809 | 0.645 ; 22.478 | 0.140 | 3.615 | 0.54 ; 24.189 | 0.185 |
| Albumin | + 1 g/dL | **0.270** | **0.094 ; 0.774** | **0.015** | **0.252** | **0.081 ; 0.782** | **0.017** |
| CRP | + 1 (log scale) | 1.416 | 0.858 ; 2.338 | 0.174 | 1.308 | 0.762 ; 2.247 | 0.330 |
| Uric acid | + 1 mg/dL | 1.116 | 0.715 ; 1.742 | 0.630 | 1.270 | 0.800 ; 2.016 | 0.312 |
| Total cholesterol | + 100 mg/dL | **4.717** | **1.680 ; 13.249** | **0.003** | **5.215** | **1.702 ; 15.974** | **0.004** |
| Monocytes | + 0.1 mm³ | 1.037 | 0.834 ; 1.289 | 0.743 | 1.132 | 0.913 ; 1.405 | 0.258 |
| Creatinine | + 1 mg/dL | 0.953 | 0.653 ; 1.391 | 0.803 | 0.839 | 0.547 ; 1.288 | 0.423 |
| Triglycerides | + 1 (log scale) | 0.658 | 0.299 ; 1.445 | 0.297 | 0.847 | 0.363 ; 1.975 | 0.701 |
| NLR | + 1 (log scale) | 1.320 | 0.696 ; 2.506 | 0.395 | 0.834 | 0.425 ; 1.637 | 0.599 |
| Serum NfL | + 1 (log scale) | 0.801 | 0.418 ; 1.534 | 0.502 | 0.851 | 0.429 ; 1.687 | 0.643 |

Abbreviations: ALSFRS-r: Amyotrophic Lateral Sclerosis Functional Rating Scale-revised; FVC: forced vital capacity; BMI: body mass index; CRP: C-reactive protein; NLR: neutrophil-to-lymphocytes ratio; NfL: neurofilament light chain.

**Supplementary Table 6. Multivariable model for variations in PD1+ Tregs, expressed as percentages (over total Tregs) or concentrations (absolute count/ul).** Multiplicative effect generalized linear mixed model with gamma distribution and log link function were employed to study PD1+ Tregs (% over total CD4 T cells) and PD1+ Tregs concentrations variations over time. Mean ratios (MR) with 95% confidence interval (CI) are reported to explain the effect of each variable on PD1+ Treg % or PD1+ Treg concentrations, respectively. Significance is set with p-values < 0.05, which are reported in bold character. The mean ratio represents the ratio between average Tregs percentage or concentrations, whichever the dependent variable is. A mean ratio equal to one implies the explanatory variable has an estimated average 0% effect on PD1+ Tregs; any increase or decrease in the mean ratio over one suggests there is an average percentage increase or decrease in PD1+ Tregs compared to reference values.

| **Variable** | | **PD1+ Tregs percentage**  **(over total CD4 T cells)** | | | **PD1+ Tregs concentration**  **(count/uL)** | | |
| --- | --- | --- | --- | --- | --- | --- | --- |
|  |  | **MR** | **95% CI** | **p-value** | **MR** | **95% CI** | **p-value** |
| Time from onset | + 30 days | **0.946** | **0.903 ; 0.992** | **0.022** | **0.946** | **0.895 ; 1.000** | **0.049** |
| ALSFRS-R | - 5 points | 1.119 | 0.922 ; 1.357 | 0.256 | 1.108 | 0.885 ; 1.386 | 0.371 |
| FVC | - 10 % | 0.999 | 0.902 ; 1.107 | 0.982 | 0.994 | 0.883 ; 1.119 | 0.919 |
| Site of onset | Bulbar vs Spinal | 0.712 | 0.295 ; 1.717 | 0.449 | 0.479 | 0.164 ; 1.397 | 0.178 |
| BMI | + 1 kg/m² | 1.047 | 0.807 ; 1.359 | 0.729 | 1.045 | 0.767 ; 1.424 | 0.780 |
| Weight change | - 10 kg | 0.891 | 0.294 ; 2.699 | 0.838 | 1.045 | 0.281 ; 3.890 | 0.947 |
| Age at onset | + 10 years | 1.169 | 0.856 ; 1.597 | 0.326 | 1.145 | 0.783 ; 1.674 | 0.484 |
| Sex | M vs F | 1.261 | 0.412 ; 3.853 | 0.685 | 1.300 | 0.342 ; 4.940 | 0.700 |
| Albumin | + 1 g/dL | 1.026 | 0.528 ; 1.995 | 0.939 | 0.924 | 0.426 ; 2.003 | 0.841 |
| CRP | + 1 (log scale) | **1.383** | **1.028 ; 1.860** | **0.032** | 1.311 | 0.938 ; 1.833 | 0.112 |
| Uric acid | + 1 mg/dL | 0.916 | 0.723 ; 1.159 | 0.464 | 1.023 | 0.779 ; 1.344 | 0.870 |
| Total cholesterol | + 100 mg/dL | 1.179 | 0.632 ; 2.199 | 0.604 | 1.338 | 0.653 ; 2.743 | 0.426 |
| Monocytes | + 0.1 mm³ | 0.899 | 0.799 ; 1.012 | 0.078 | 0.969 | 0.844 ; 1.114 | 0.662 |
| Creatinine | + 1 mg/dL | 1.188 | 0.975 ; 1.447 | 0.088 | 1.110 | 0.876 ; 1.405 | 0.387 |
| Triglycerides | + 1 (log scale) | **1.784** | **1.085 ; 2.931** | **0.022** | **2.243** | **1.266 ; 3.972** | **0.006** |
| NLR | + 1 (log scale) | **1.572** | **1.042 ; 2.371** | **0.031** | 0.976 | 0.607 ; 1.571 | 0.921 |
| Serum NfL | + 1 (log scale) | **1.495** | **1.010 ; 2.213** | **0.044** | **1.600** | **1.009 ; 2.538** | **0.046** |

Abbreviations: ALSFRS-r: Amyotrophic Lateral Sclerosis Functional Rating Scale-revised; FVC: forced vital capacity; BMI: body mass index; CRP: C-reactive protein; NLR: neutrophil-to-lymphocytes ratio; NfL: neurofilament light chain.

**Supplementary Table 7. RAP-ALS study group**

| **RAP-ALS sites and locations** | **Investigators** |
| --- | --- |
| ALS Center, Azienda Ospedaliero Universitaria di Modena, Modena, Italy | Jessica Mandrioli  Elisabetta Zucchi  Ilaria Martinelli  Cecilia Simonini  Giulia Gianferrari  Nicola Fini  Roberta Bedin  Annalisa Gessani  Francesca Prompicai  Silvia Parisi  Daniela Gallesi  Andrea Ghezzi |
| Statistical Unit of Modena, University of Modena and Reggio Emilia, Italy | Roberto D’Amico  Federico Banchelli  Riccardo Cuoghi Costantini |
| Immunology Lab, University of Modena and Reggio Emilia, Italy | Andrea Cossarizza  Sara De Biasi  Domenico Lo Tartaro  Anita Neroni  Marcello Pinti  . |
| ALS Center, Maggiore della Carità Hospital, University of Piemonte Orientale, Novara, Italy | Letizia Mazzini  Ada Scognamiglio  Fabiola De Marchi  Paola Odoli |
| Centro Regionale Esperto per la SLA (CRESLA), Azienda Ospedaliero Universitaria Città della Salute e della Scienza, University of Turin, Turin, Italy | Adriano Chiò  Andrea Calvo  Cristina Moglia |
| IRCCS Istituto Neurologico Carlo Besta di Milano, Milan, Italy | Giuseppe Lauria Pinter  Eleonora Dalla Bella  Raffaella Lombardi  Enrica Bersano |
| NEuroMuscular Omnicentre (NEMO), Fondazione Serena Onlus, Milan, Italy | Christian Lunetta  Claudia Tarlarini  Francesca Gerardi  Valeria Sansone |
| Neurological Clinic, IRCCS Ospedale Policlinico San Martino, Genoa, Italy | Claudia Caponnetto  Giuseppe Meo  Corrado Cabona  Chiara Gemelli |
| Centro per la diagnosi, cura e ricerca nella SLA e Malattie del Motoneurone, Azienda Ospedale Università di Padova, Padua, Italy | Gianni Sorarù  Andrea Fortuna |
